# Effect of a Low Glycemic Index/Slow Digesting (LGI/SD) Carbohydrate Product on Maternal Glycemia and Neonatal Body Composition in Obese Pregnant Women: The NIGOHealth Randomized Clinical Trial

**DOI:** 10.3390/nu17111942

**Published:** 2025-06-05

**Authors:** Mercedes G. Bermúdez, María García-Ricobaraza, José Antonio García-Santos, M. Teresa Segura, Alberto Puertas-Prieto, José Luis Gallo-Vallejo, Carmen Padilla-Vinuesa, Berthold Koletzko, Geraldine E. Baggs, Elena Oliveros, Ricardo Rueda, Cristina Campoy

**Affiliations:** 1 Department of Pediatrics, School of Medicine, University of Granada, Avda. de la Investigación 11, 18016 Granada, Spain; mariaricobaraza@ugr.es (M.G.-R.); joseantonio_gsantos@outlook.es (J.A.G.-S.); maite.segura.moreno@gmail.com (M.T.S.); 2 Instituto de Investigación Biosanitaria ibs.GRANADA, Health Sciences Technological Park, 18012 Granada, Spain; 3 Department of Obstetrics & Gynaecology, Hospital Universitario Virgen de las Nieves, Avda. Fuerzas Armadas s/n, 18014 Granada, Spain; apuertas51@hotmail.com (A.P.-P.); joselgallov@ugr.es (J.L.G.-V.); 4 Gynaecology and Obstetrics Unit, Hospital Universitario Clínico San Cecilio, Avda. Conocimiento, s/n, 18016 Granada, Spain; mc.padilla.sspa@juntadeandalucia.es; 5 Department of Obstetrics and Gynecology, Faculty of Medicine, University of Granada, Avda. Investigación 11, 18016 Granada, Spain; 6 Department of Paediatrics, LMU—Ludwig Maximilians Universitaet Munich, Dr. von Hauner Children’s LMU University Hospital, 80337 Munich, Germany; office.koletzko@med.uni-muenchen.de; 7 German Center for Child and Adolescent Health, 80337 Munich, Germany; 8 Abbott Nutrition R&D, Columbus, OH 43219, USA; geraldine.baggs@abbott.com; 9 Abbott Nutrition R&D, Abbott Laboratories, 18004 Granada, Spain; elena.oliveros@abbott.com (E.O.); ricardo.rueda@abbott.com (R.R.); 10 Spanish Network of Biomedical Research in Epidemiology and Public Health (CIBERESP) (Granada’s Node), Institute of Health Carlos III, 28029 Madrid, Spain

**Keywords:** obese pregnant women, gestational diabetes mellitus, low glycemic index/slow digesting (LGI/SD) carbohydrate product, AUC, HbA1c, insulin, early programming

## Abstract

**Background/Objectives:** Obesity during pregnancy is strongly related to increased insulin resistance, and subsequent development of metabolic syndrome-like disorders, such as glucose intolerance, pre-eclampsia, as well as preterm birth, and cesarean delivery. Nutrition can influence the evolution of glycemic response and may help improve adverse pregnancy outcomes and long-term complications. The main objective of the Nutritional Intervention during Gestation and Offspring Health (NIGOHealth) randomized clinical trial (ClinicalTrials.gov Identifier: NCT02285764) was to investigate the potential effects of a low glycemic index/slow digesting (LGI/SD) carbohydrate product on maternal glycemia (glucose AUC at 27^+0^–28^+6^ weeks; maternal fasting blood glucose (MFBG) at 34^+0^–36^+0^ weeks), and neonatal body composition. **Methods:** Obese pregnant women were randomized: 230 in the intervention group (IG), who consumed two servings of an LGI/SD study product daily from 15 weeks of pregnancy until delivery, and 102 participants in the Standard of Care (SOC) group. **Results:** When analyzing baseline characteristics, significant differences were found in glucose metabolic parameters with higher values for IG than for the SOC group, compromising the group’s comparability. Despite this, a statistical analysis was conducted (intention-to-treat analysis/evaluable cohort): no differences were detected regarding maternal blood glucose AUC at 27^+0^–28^+6^ weeks, nor for MFBG at 34^+0^–36^+0^ weeks. Nonetheless, HbA1c (%) at 34^+0^–36^+0^ weeks was significantly lower in the IG vs. the SOC group (5.26 ± 0.03, 5.31 ± 0.04, *p* = 0.007) after adjusting for baseline conditions. **Conclusion**: This result might suggest a potential effect of the intervention on Evaluable participants. However, it should be taken with caution, due to the limitations of the study. More RCTs should be carried out to explore the effects of LGI/SD products on glycemic response in obese pregnant women.

## 1. Introduction

Obesity in women who enter pregnancy predicts short- and long-term adverse health outcomes for both mother and offspring, because maternal obesity can propagate intergenerational cycles of increasing obesity and diabetes [1]. Globally, overweight and obesity prevalence rates have been increasing steadily, now reaching epidemic proportions worldwide [2]. In pregnant women, the prevalence of both conditions has also increased dramatically in both high- and middle-income countries [3]. The prevalence of obesity among women of reproductive age varies across high-income countries [4]. Estimations of obesity among pregnant women in 23 EU countries suggest that UK women have the highest prevalence of obesity in Europe (25.2%), and those from Poland the lowest (7.1%) [5]. Conversely, in the United States, around 55% of reproductive-age women are overweight/obese [6].

Obese women have increased insulin resistance (IR), insulin response, and inflammatory cytokines compared with average-weight women both before and during pregnancy. [7]. Maternal IR is a normal part of human pregnancy and is critically important to maintain the maternal fuel supply to support the growing fetus, particularly during the third trimester [8]. In normal pregnancies, glucose homeostasis is maintained despite adaptation towards IR, which is required for the regulation of maternal energy metabolism and fetal growth [9]. However, women with obesity or a history of gestational diabetes mellitus (GDM) enter pregnancy with preexisting IR that worsens with advancing gestation. Overweight and obesity during pregnancy are strongly related to exacerbated IR [10] and the subsequent development of metabolic syndrome-like disorders during pregnancy, such as hypertension [11], hyperlipidemia, glucose intolerance [12], GDM [13], and coagulation disorders [7], as well as pre-eclampsia, preterm birth, and cesarean delivery [14].

In addition, infants and children of women who are obese and develop IR may be more likely to develop metabolic complications due to excess glucose exposure in the intrauterine environment and are at risk of increased adiposity and obesity later in life [15]. Deregulated glucose metabolism during pregnancy has been shown to result in increased macrosomia (birth weight > 4 kg) and infants born large for gestational age (LGA) [16], even in non-diabetic population, and may also cause a variety of pregnancy complications, including increased prenatal and perinatal mortality, perinatal complications [6], and neurodevelopmental delay [17]. Moreover, multiple studies have reported that children of women with GDM have a greater prevalence of childhood obesity and glucose intolerance, even at glucose concentrations lower than those currently used to define GDM, compared to normoglycemic women [18].

On the other hand, nutrition, especially carbohydrates (CHO) and fiber, contributes to regulating glycemic response and can, therefore, influence the establishment and evolution of GDM, as well as the risk of clinical outcomes for both the mother and the infant [19]. Consuming a high-fiber and/or low-glycemic index (LGI) diet late in pregnancy may help to blunt the mid to late pregnancy-related increases in IR [20], which are exacerbated in obese pregnancies [21]. Due to the potential effects of oral hypoglycemic medications on fetal growth, nutrition may be a key tool for the prevention and management of GDM. In particular, low-glycemic index/slow-digesting (LGI/SD) foods operate on the principle that they are digested and absorbed more slowly and, hence, help moderate the post-prandial increase in blood glucose concentrations. LGI foods have been used to ameliorate the post-prandial glucose and insulin response in pregnancy in numerous interventional studies [22,23,24,25,26,27]. Results from a recent meta-analysis showed that intervention with low-GI diet advice might reduce gestational weight gain (GWG) and lower the risk of preterm delivery in pregnant women at high risk of GDM, especially in obese women [28]. On the other hand, Calancie et al. recently published a review describing interventions conducted in pregnant women with risk factors for hyperglycemia, indicating that interventions initiated in early pregnancy (<20 weeks) can reduce the risk of excess neonatal adiposity and macrosomia [29]. Therefore, strategies supporting enhanced glucose regulation in those with impaired glucose tolerance during pregnancy may help improve adverse pregnancy outcomes and long-term complications.

The main objective of the Nutritional Intervention during Gestation and Offspring Health (NIGOHealth) RCT was to investigate the potential effects of an LGI/SD nutritional product for obese pregnant women to improve maternal glycemic outcomes [maternal blood glucose area under the curve (AUC) at the second trimester (27^+0^–28^+6^ weeks) with a 2 h 75 g oral glucose tolerance test (OGTT)]. Moreover, secondary variables included neonatal body composition ≤ 48 h after birth (PEA POD^®^), and maternal fasting blood glucose (MFBG) at V3.

This study was part of the EarlyNutrition project (FP7-289346-EarlyNutrition), funded by the European Commission’s 7th Framework Program and Abbott Nutrition.

## 2. Materials and Methods

### 2.1. Study Overview

The NIGOHealth study (ClinicalTrials.gov Identifier: NCT02285764) was designed as an open (neither the investigators, medical staff, nor outcome evaluators were blinded to the identity of the study treatment), prospective, randomized nutritional supplementation study to determine if an LGI/SD supplement with nutritional guidance can improve glycemia and glycemic parameters in obese pregnant women compared to the Standard of Care (SOC) provided.

Recruitment (screening) took place at ≤14^+6^ weeks, and the first study visit (V1) took place at the end of the first trimester of pregnancy (15^+0^–16^+6^ weeks; median: 15.71 weeks). Study V2 and V3 were held at 27^+0^–28^+6^ weeks and 34^+0^–36^+0^ weeks of gestation [27.78 and 34.86 weeks (median values)], respectively. Maternal age, ethnicity, weight, BMI, and parity were recorded at the screening visit, taken from medical records, self-reported, or as a first-trimester measurement, and checked again at V1. In addition, socioeconomic status, smoking, and alcohol use were registered as well. After the ICF was signed and dated, a routine 75 g oral glucose tolerance test (OGTT) was performed. After filling out questionnaires about physical activity (IPAQ), health status (EQ-5D), food frequency questionnaire (FFQ), and Edinburgh Postnatal Depression Scale (EPDS), participants were randomized to the IG or to the SOC study groups. The intervention involved dietary counseling, physical activity recommendations, as well as the study product (SP) provision after randomization in V1.

**Figure 1:**
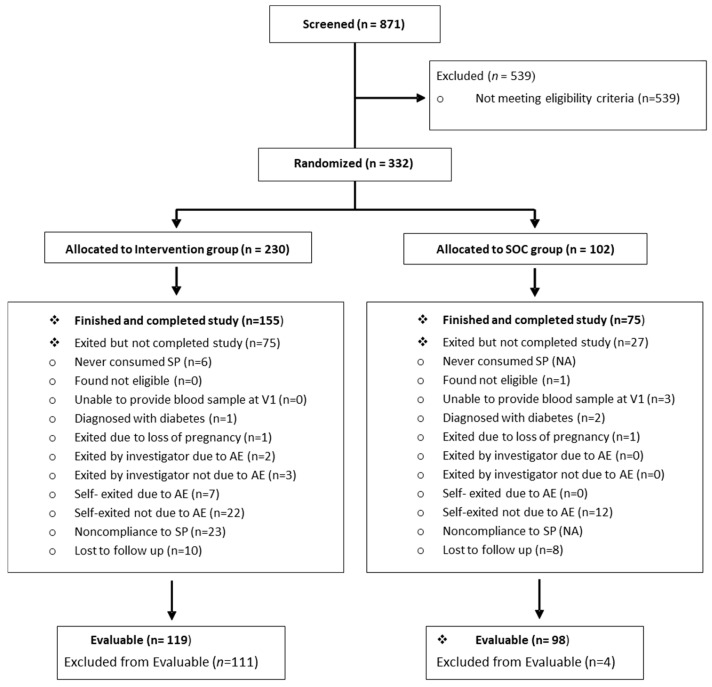
Participant flowchart, with details about the number of participants allocated to each arm of the RCT, and reasons for dropouts and exclusions. AE: Adverse Events; NA: Not Applicable; SOC: Standard of Care; SP: Study Product; V1: Visit 1.

Target enrollment was a total of 324–363 obese pregnant women (enrolled in a ratio of approximately 2:1 in the intervention and SOC groups, respectively) from study centers in Spain and Germany. A detailed participant flowchart with details about the number of participants allocated to each arm of the RCT, and reasons for dropouts and exclusions, is shown in Figure 1. A total of 871 women were assessed for eligibility; 539 individuals (61.88%) did not meet inclusion criteria and, consequently, were excluded. A total of 332 participants were finally recruited; 230 participants were allocated to the IG, and 102 to the SOC group.

### 2.2. Study Protocol

#### 2.2.1. Participants

Obese pregnant women aged ≥18 years with a pre-pregnancy or screening visit (≤14^+6^ weeks of gestation) body mass index (BMI) ≥ 30 kg/m^2^ and a singleton pregnancy were screened for the NIGOHealth RCT. Women were willing to consume 2 servings of SP per day from V1 until delivery (if randomized to IG), follow appropriate nutritional guidance, refrain from consuming other caloric nutritional supplements that were not approved by the study staff, if applicable, and voluntarily signed and dated an informed consent form (ICF) prior to any participation in the study. Finally, participants were willing to provide body composition measures for their infants at birth.

The following exclusion criteria were established: adverse maternal and/or fetal medical history expected to alter blood glucose regulation; previously diagnosed diabetes, previous GDM, systemic lupus erythematosus, anti-phospholipid syndrome, known renal disease, treated hypertension, uncontrolled hypothyroidism and/or cancer; use of prescription medications that could impact blood glucose, or injected/oral corticosteroids at the discretion of the study physician; previous bariatric surgery; lactose intolerance or allergy to milk protein, soy or other ingredients in the product, and/or required a special dietary regime; or taking part in another clinical trial that could impact participation in this study.

The study was conducted in accordance with the Declaration of Helsinki and approved by the Institutional Review Board (or Ethics Committee) of the Mother–Infant University and San Cecilio University Hospitals in Granada (Spain), as well as at Ludwig Maximilians Universitaet Munich, Dr. von Hauner Children’s Hospital (Germany)/(approval date 30 November 2015). Screening and recruitment process spanning from December 2014 through September 2017 in the above-mentioned medical centers.

Upon fulfilling the inclusion criteria and receiving written informed consent from the subject prior to any study participation, participants were randomized to the IG or to the SOC group. Randomization was undertaken using a secure web-based data management system (MedSciNet AB-U.K. Ltd., Reading, UK). The randomization schedule used a dynamic minimization algorithm, which included maternal ethnicity, age, BMI, and parity as stratification factors to avoid bias between study groups. The initial study design showed a recruitment ratio of 1:1; however, due to the higher dropout of participants in the IG arm during the recruitment period until the full completion of the protocol, randomization to IG or SOC was changed into a 3:1 ratio to achieve an unequal allocation of 2:1 (IG:SOC) by the end of the trial.

#### 2.2.2. Nutritional Intervention

Participants randomized to the IG were instructed to consume one 237 mL TetraPak© container of SP 2 times per day; once in the morning with breakfast, and once in the afternoon as a stand-alone snack between lunch and dinner, ideally 2–3 h before dinner, starting the day after V1 (15^+0^–16^+6^ weeks) till V4 (birth; estimation 37–40 weeks gestation). Approximate study product composition is shown in Supplementary Table S1. Participants in the IG received dietary recommendations for pregnancy based on recognized national standards, and instructions on how to incorporate the study beverage into those recommendations without causing an extra calorie burden, including a daily meal plan example. They were also instructed to continue taking the product until delivery, recording product consumption. Adherence to the study was self-reported through a Product Intake Record, reviewed at Study V2 and V3, and returned to the study site at V4. Moreover, participants were asked to return any product not taken, so the research staff could check the amount of tetrapacks consumed.

Participants in the SOC group received only what is normally provided at each obstetric practice site where they receive prenatal care, which includes routine prenatal visits with an obstetrician: history and physical exam, monitoring of weight and blood pressure, fetal ultrasound and heart tones, and routine blood and urine analyses. They did not receive any study-specific information on diet, nutrition, exercise, or pregnancy weight gain.

All the women participating in the NIGOHealth study took a daily supplement of 400 µg folic acid and 200 µg of iodine throughout pregnancy, according to international medical recommendations [30].

#### 2.2.3. Assessment of Outcomes

Table 1 details the schedule of study assessments. At each study visit, maternal measurements, including weight, height, skinfold thickness, blood pressure, pulse, and BMI were used to determine maternal anthropometry.

At visits 1, 2, and 3, participants had blood drawn by venipuncture after an overnight fast of 10 h. Maternal blood samples from V1, V2, V3, and V4, as well as the umbilical cord blood, were immediately sent for analysis of hematologic and biochemical markers at the hospital laboratory, including serum biomarkers [glucose, cholesterol, triglycerides, HbA1c, insulin, and homeostasis model assessment of insulin resistance (HOMA-IR). HOMA-IR, a measure of insulin resistance [31,32], was validated as an estimate of insulin resistance during early, mid-, and late pregnancy [33].

OGTT at V1 and V2 were performed in all mothers using the International Association of Diabetes Pregnancy Study Group (IADPSG) criteria [34]. Blood samples were extracted at fasting state, and 1 h and 2 h post-75 g glucose load.

Weight, length, head and abdominal circumferences, skinfold measurements, and air displacement plethysmography (PEA POD^®^, COSMED SRL, Rome, Italy) were used to determine newborn anthropometry and body composition. Moreover, gestational age at delivery (weeks + days) and newborn sex were registered at V4 (parturition).

#### 2.2.4. Statistical Analysis

All analyses used the intention-to-treat (ITT) principle, classifying each subject participant according to her randomly assigned group, regardless of duration or compliance. An ITT analysis was considered the primary analysis for the study. All available data from participants and infant pairs whose mothers received at least one study feeding of the maternal supplement in the IG and/or enrolled in the trial in the SOC group were included in the modified ITT analysis.

The secondary analysis dataset included only data from those participants determined to be Evaluable. Participant outcome data were classified as “Evaluable” for the analysis until one or more of the events described below occurred:

Subject

1. Subject did not provide OGTT data at Visit 2.
2. The participants in the IG had <75% average intake of the SP between Visits 1–2, as determined by Product Intake Records.
3. The participants in the IG had <50% intake of the SP in the 7 days prior to Visit 2, as determined by the Product Intake Records.
4. The window between Visit 1 and Visit 2 was <10 weeks.
5. The participant had a diabetes diagnosis at V1 from fasting glucose ≥ 126 mg/dL.

Infant

1. The infant was born at <37^+0^ or >41^+6^ weeks gestation.
2. The infant had a major congenital disease that would impact intrauterine growth, as determined by the study physician.
3. Maternal subjects in the IG had <75% average intake of the SP between Visits 1–4, as determined by the Product Intake Records.
4. The maternal participant was not Evaluable.

All tests were 2-tailed with a significance level of α = 0.05. SAS software (version 9.2; SAS Institute, Cary, NC, USA) was used for all computations. Baseline measurements were compared between treatment groups by ANOVA for normally distributed variables, Wilcoxon rank sum test for non-normal continuous variables, and Chi-square or Fisher test for categorical variables. Infant characteristics at delivery and postnatal infant measurements were compared similarly. Infant variables at delivery were adjusted by ANCOVA (factor: study group, covariates: maternal ethnicity, age, BMI, parity). Maternal anthropometric measurements and metabolic parameters were obtained at baseline and V3. The total area under the blood glucose curve (AUC) and adjusted (incremental) area under the curve (AAUC) after two hours of OGTT at Visit 2 were calculated using the trapezoidal rule [35]. AAUC was calculated by subtracting from the total AUC the area below the value at time-zero, or equivalently by subtracting the time-zero value from the sample points before calculating the AUC. Overall changes in weight, BMI, body composition, and blood pressure (V3–baseline) were compared between treatment groups by ANCOVA with different confounders. Changes in metabolic variables, many of which were markedly skewed in distribution, were compared and adjusted by ANCOVA (study group, maternal ethnicity, age, BMI, parity), which is robust to non-normality. Curve slopes were obtained as solutions to fixed effects of time and time*treatment group in mixed-effects models using the NOINT option in the MODEL statement and treating time as a continuous variable. The slopes represented the rate of change in the response over time for each of the treatment groups.

The sample size was estimated using the software nQuery^®^ Advisor 5, based on variability in blood glucose AUC after 2 h of ingesting a 75 g glucose challenge, in similar study populations. Based on the analysis of the HAPO study [36], where about a 5% difference in AUC was associated with an increased positive predictive value (PPV) of infant birth weight and body fat > 90th centile, achieving a 5% difference (mean = 37.5, standard deviation = 99.8, effect size = 0.376) in AUC was considered clinically meaningful. To reach a statistical power of 80%, using a two-sided 0.05 level *t*-test, the sample size would be 254, split unequally between the two groups (2:1 allocation), with 169 in the LGI/SD and 85 in the SOC groups, respectively. Assuming an attrition rate between 21.6 and 30%, enrollment of 324 to 363 participants was targeted.

## 3. Results

### 3.1. Demographic and Other Baseline Characteristics

As shown in Figure 1 and Table 2, 332 women were finally enrolled and randomly assigned to the IG (*n* = 230) or the SOC group (*n* = 102). Participants represented a moderately high-risk obstetric population of obese pregnant women, with 28.92% (96/332) considered to have advanced maternal age (≥35 years old). They were mostly of Spanish origin and had studied in high school or university. No differences were found at baseline between study groups regarding maternal age, parity, or ethnicity (Table 2).

In the case of demographic and lifestyle parameters, there were more employed participants in the IG (*p* < 0.001).

Regarding maternal anthropometry, no significant differences were detected between both groups of participants. However, concerning vital signs, the average arm diastolic blood pressure was significantly higher in the SOC group (*p* = 0.012).

One determinant finding regarding baseline characteristics is that significant differences were found in key glucose metabolic parameters [fasting glucose (*p* < 0.001), insulin (*p* = 0.044), and HOMA-IR (*p* = 0.010)] (Table 2), with higher values for the IG than for the SOC group, thus compromising groups’ comparability. This fact is of great importance considering that the main variable and one of the secondary variables are directly related to glucose metabolism.

### 3.2. Main Outcome: AUC for Maternal Blood Glucose at 28 Weeks of Gestation

As stated above, significant baseline differences were found in key glucose metabolic parameters, with higher values for the IG than for the SOC group, compromising the groups’ comparability. Despite those differences, a statistical analysis was conducted (ITT/Evaluable cohort) for the primary and secondary variables.

**Figure 2:**
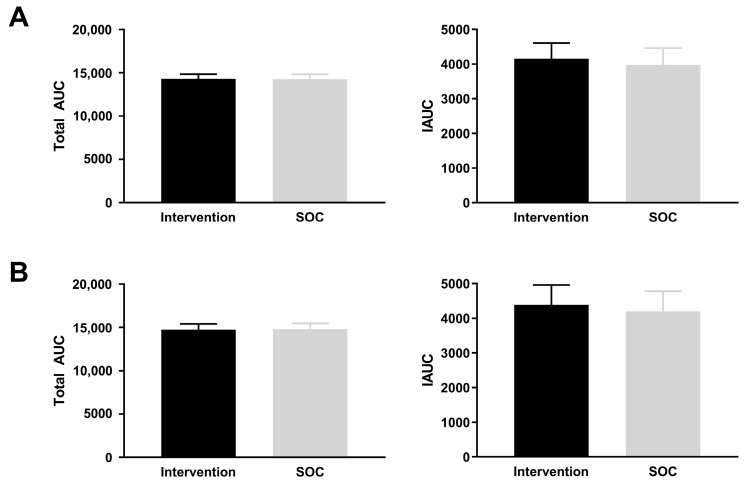
Total Area Under the Curve (AUC) and Incremental AUC (IAUC) at Visit 2 for intervention and SOC groups in the ITT cohort (**A**), and in the Evaluable cohort (**B**). ANCOVA included covariate maternal HbA1c at baseline, and factors Study Group, maternal ethnicity, age, BMI, and parity.

The main variable, AUC at 28 weeks of gestation at V2, was compared for the IG vs. SOC group. The analyses of total AUC show that the effect of the SP intervention was not statistically significant in the ITT (*p* = 0.89) or the Evaluable cohort (*p* = 0.89). Neither were differences in IAUC detected in the ITT or in the Evaluable cohort (*p* = 0.56; *p* = 0.57, respectively) (Figure 2A,B).

### 3.3. Secondary Variable: MFBG at 34^+0^–36^+0^ Weeks of Pregnancy

Regarding MFBG at V3 (34^+0^–36^+0^ weeks), no statistically significant differences between groups (IG: 83.05 ± 0.72; SOC: 79.87 ± 0.99; *p* = 0.011; adjusted *p* = 0.050) were detected in the ITT cohort; in the Evaluable cohort, statistical significance was lost in the model adjusted for confounding factors (Table 3).

Nonetheless, due to statistically significant baseline differences between the IG and SOC groups, further analysis aimed to determine whether the study intervention had an impact on other glucose metabolism markers, such as MFBG, insulin, HOMA-IR, and HbA1c, was performed throughout the study period.

Those additional analyses of the key glucose markers were carried out by adjusting for HbA1c plus the variable itself (MFBG, HOMA-IR, or insulin) at V1 and comparing results between the IG and SOC groups. Maternal HbA1c at V3 was significantly lower for the IG compared with the SOC group (*p* = 0.007 in the Evaluable cohort). No other differences in MFBG, HOMA-IR, or insulin were detected (Table 3).

**Figure 3:**
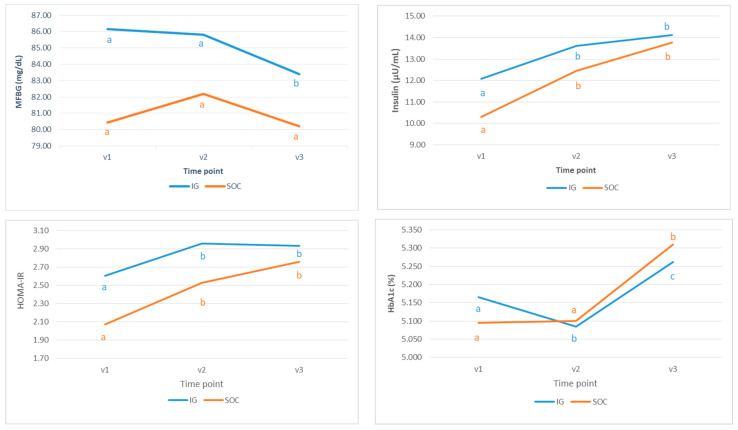
Evolution of MFBG (mg/dL), Insulin (µU/mL), HOMA-IR, and Hemoglobin A1c (HbA1c) throughout the study period V1 (15^+0^–16^+6^ weeks), V2 (27^+0^–28^+6^ weeks), and V3 (34^+0^–36^+0^ weeks) for each group in the Evaluable cohort. Different letters (a, b, c) at study visits indicate that averages are statistically different (*p* < 0.05) within the group.

Additionally, the evolution of glucose markers throughout the study period in each group separately was analyzed, and the results are shown in Figure 3. MFBG at V3 was significantly lower in IG than in V2 and V1. In contrast, no differences were detected in the SOC group. Insulin and HOMA-IR V2 and V3 values were significantly higher than the value at V1, both in the IG and SOC group, and a significant increase in insulin from visit 2 to visit 3 in the SOC group, but not in the IG, was detected.

For HbA1c, a significant decrease in V2 was observed for those in the IG. In the SOC group, the value at V3 was significantly greater than at V1 and V2 (Figure 3). HbA1c reflects long-term glycemic exposure, representing the average glucose concentration over the preceding 8–12 weeks [37].

Moreover, a statistical comparison between curve slope variation for each biomarker was performed. For the Evaluable cohort, the HbA1c slope for evolution in the IG was significantly lower compared to that of the SOC group. No significant differences in slopes between treatment groups for MFBG, HOMA-IR, and insulin were found when the model was adjusted for age, BMI, ethnicity, and parity, with (Table 4) or without the covariate baseline HbA1c.

### 3.4. Secondary Variable: Neonatal Body Composition

Concerning neonatal body composition ≤ 48 h after birth, differences in fat mass, body mass, and body volume, as well as head and abdominal circumferences, were detected in the Evaluable cohort, being higher in the IG compared to SOC, although significance was lost after model adjustment for confounding factors (Table 5).

On the other hand, no differences in characteristics at delivery (maternal gestational weight gain, gestational age at delivery, mode of delivery, SGA and LGA conditions, sex, and APGAR score) were detected in the ITT cohort nor in the Evaluable cohort of the babies from NIGOHealth study (Supplementary Table S2).

### 3.5. Additional Analysis

As shown above in Figure 3, MFBG at baseline was significantly higher in the IG, and, according to the IADPSG recommendations [38], a fasting plasma glucose range of 5.1 to 6.9 mmol/L (~91.8–124.2 mg/dL) before 24 weeks of gestation defines early intermediate hyperglycemia or early GDM (eGDM) [39,40]. Therefore, there might be more participants predisposed to suffer from GDM in the IG than in the SOC group, as was finally confirmed in the study results at V2: 32% (*n* = 52) vs. 16% (*n* = 13), respectively (*p* = 0.002). Moreover, those participants who ultimately developed GDM later during the study had worse glucose parameters at V1 than participants who did not (Supplementary Table S3). Hence, additional statistical analysis was performed, including only those participants from each group (IG and SOC) who had an MFBG below 92 mg/dL at V1, aiming to exclude all participants who may have a certain predisposition to develop GDM and may jeopardize study intervention evaluation. As shown in Table 6, maternal HbA1c at V2 and V3 were significantly lower in the IG compared to the SOC group. There were no other significant differences in AUC, AAUC, or glucose parameters (MFBG, insulin, and HOMA-IR) between groups at V2 and V3.

Regarding neonatal body composition and anthropometry measures, no significant differences between infants in the IG vs. SOC group were detected (Table 6).

Further analyses were performed comparing the effects of the nutritional intervention on maternal and offspring outcomes in participants who finally did not develop GDM or those who suffered from it. In this regard, baseline glucose parameters (blood glucose AUC, glucose, insulin, HbA1c, and HOMA) in participants with GDM and non-GDM were significantly different, as expected (Supplementary Table S3). With respect to AUC, MFBG, or neonatal body composition in participants who developed GDM, there were no differences between groups in the ITT cohort or in the Evaluable cohort (Supplementary Table S4A–C). However, among those participants who did not develop GDM, significant differences were observed in HbA1c at V2 and V3 (ITT cohort), and insulin levels at V2 (Evaluable cohort) (Supplementary Table S4A). No significant differences were detected in the ITT cohort, nor in the Evaluable cohort regarding neonatal body composition in non-GDM participants (Supplementary Table S4B,C).

### 3.6. Adverse Events (AEs)

Non-serious and serious AEs were reported for 41 (40.2%) participants in the SOC group, and 105 (45.7%) in the IG. Non-serious AEs were reported for 40 participants (39.2%) in the SOC group and 97 participants (42.2%) in the IG. The greatest number of participants had GDM (22.6% in the IG and 12.7% in the SOC group), and events related to the gastrointestinal disorder system organ class included 8 (7.8%) participants in the SOC group and 51 (22.2%) in the IG (Supplementary Table S5). The most commonly reported were dyspepsia (i.e., heartburn) and diarrhea. Other AEs reported were anemia (4.8% in the IG and 15.7% in the SOC group) and hyperlipidemia (7.0% in the IG and 12.7% in the SOC group). Serious AEs were reported for 2 participants (2.0%) in the SOC group (late abortion, and HELLP syndrome) and for 11 participants (4.8%) in the IG (including fetal death, TOP after the fetus was diagnosed with tetralogy of Fallot, premature delivery, severe pre-eclampsia, among others). Causality was assessed if SP was consumed at any time within the 48 h prior to the onset of an event. The majority of AEs were deemed not related or probably not related to SP intake by the study physician. There were 89 (70.6%) events not related to SP, while those categorized as probably not related were 26 (20.6%). Events categorized as possibly related to SP were only 10 (7.9%), and, finally, there was a single event (0.8%) categorized as probably related to SP.

Regarding infants born to mothers participating in the study, non-serious AEs (such as hypoglycemia, respiratory distress, growth restriction, …) were reported for two infants (2.0%) born to mothers in the SOC group, and in five infants (2.2%) born to mothers in the IG (Supplementary Table S5). Serious AEs were reported for six infants (2.6%) born to mothers only in the IG: neonatal asphyxia; bradycardia; septic shock upon birth; and meconium aspiration syndrome. All six (100.0%) of the SAEs reported from infants born to mothers in the IG were deemed not related to SP intake by the study physician.

## 4. Discussion

The main objective of the NIGOHealth RCT was to investigate the potential effects of an LGI/SD nutritional product for obese pregnant women on maternal glycemia and neonatal body composition. Regardless of the randomization schedule followed in the study designed to minimize possible differences between study groups, significant baseline differences were found in key glucose metabolic parameters, which may have compromised the groups’ comparability. Despite this, a statistical analysis was conducted, and no significant differences were observed in the primary variable, blood glucose AUC at V2, comparing IG vs. SOC group, in either Evaluable or ITT cohorts. In relation to the secondary variable, MFBG at V3, results showed no significant differences between groups. Nevertheless, considering those metabolic differences at baseline, another set of analyses was performed to assess the impact of the SP on the evolution of some glucose metabolism markers throughout the study period. In this regard, no significant differences were found in MFBG, HOMA-IR, or insulin values at V2 or V3. Nevertheless, maternal HbA1c at V3 was significantly lower for the IG compared with the SOC group in the Evaluable cohort.

Notably, results showed that participants in the IG had a more favorable evolution of HbA1c than the SOC group throughout the study period when the groups were evaluated separately: lower values of maternal HbA1c at V3 in the IG were noticed when compared to the SOC group. Moreover, a significant difference was observed in the variation in HbA1c slope through the study in the IG vs. SOC group. This within-group analysis shows that although both study groups show similar trajectories, a potential effect of the SP might not be ruled out.

Regarding neonatal body composition, significant differences were found in fat mass, body mass, and body volume, being higher in the IG compared to the SOC group in the Evaluable cohort, although significance was lost after model adjustment for confounding factors. Other studies observed a lower birth weight among infants of women consuming an LGI diet [41,42,43]. In the current study babies born to mothers consuming the LGI/SD product were heavier and taller compared to the SOC group, although differences do not reach statistical significance. In this regard, results from a meta-analysis [28] of different RCTs [22,23,25,26,44,45] reported no significant differences in birth weight and length, when comparing babies born to obese women receiving LGI diet advice on pregnancy outcomes.

Results in the current study showed bigger abdominal circumference (AC) in those babies born to IG mothers, both in the ITT and Evaluable cohort, although significance was lost after adjusting for confounding factors. Larger AC at birth may reflect visceral adiposity [46] and, together with birth weight, are strongly associated with adverse metabolic outcomes later in life [47]. Children included in the NIGOHealth study are being monitored in succeeding years to evaluate their evolution and for early signs of premature obesity. Epidemiological studies have shown that the effects of in utero exposure on metabolic health might be recognizable later in life [15], modulating, for instance, epigenetic changes in offspring genome [48]. Of note, participants in the NIGOHealth study exhibited GWG within the international recommendations for their given pre-pregnancy BMI [49].

Obesity in pregnancy is associated with a major number of complications [6,10,11,12,13,14,15]. Moreover, positive dose–response relations between maternal BMI and macrosomia and LGA have been very well documented [50]. Pharmacologic treatment of metabolic abnormalities during pregnancy has a limited role, highlighting the importance of dietary intervention. Different approaches have been reported aiming to prevent adverse consequences in obese/overweight pregnancies. Walsh et al. in the ROLO study showed that pregnant women receiving LGI diet advice had significantly lower GWG and less maternal glucose intolerance compared to those following the Standard of Care; however, the incidence of LGA infants was not reduced [45]. In the UPBEAT study [26] of obese women receiving an intensive behavioral intervention to increase physical activity and improve diet quality, there were improvements in GWG, although no other additional benefits were observed. Phelan et al. reported the effect of a behavioral lifestyle intervention with partial meal replacement compared to usual care on weekly GWG rate, cardiovascular disease risk factors, and the incidence of pregnancy complications: a trend in reductions in fasting glucose and systolic blood pressure from the study entry to 35–36 weeks of gestation was detected when compared with the enhanced usual care [51]. In the RADIEL study, women with a history of GDM and/or obesity receiving individualized counseling on diet, physical activity, and weight control, had lower incidences of GDM and GWG [52]. Zhang et al. reviewed the effects of LGI diets on all pregnant women, both those having healthy pregnancies at risk for GDM and those with GDM. Pregnant women following LGI diet advice had beneficial effects on maternal outcomes (MFBG, and post-prandial glucose levels), although there were no significant benefits on newborn outcomes (macrosomia, prematurity, or HC or AC) [24]. In the LIMIT trial, babies born to obese or overweight women in the intensive lifestyle arm with advice to reduce intake of refined carbohydrates, were less likely to be LGA, to have respiratory distress syndrome, and had shorter hospital stays [53]. Calancie et al. recently published a review describing interventions conducted in pregnant women with risk factors for hyperglycemia [29], including overweight and/or obesity, with interventions including diet only [54,55], physical activity or exercise only [56,57], diet and physical activity or exercise combined [25,58], lifestyle counseling and mixed interventions [53,59,60,61,62], in early pregnancy (<20 weeks) that may improve maternal and neonatal outcomes.

LGI foods have been used to ameliorate the post-prandial glucose and insulin response in pregnancy in numerous interventional studies [22,23,24,25,26,27]. A Preliminary Pilot Study [63] prior to the current NIGOHealth study was conducted to test the effect of a nutritional product on glycemia and insulinemia in obese pregnant women This study showed differences in numerous parameters of glycemic control while consuming the LGI/SD carbohydrate supplement vs. control product or habitual diet: a significant improvement in overall daytime glucose blood levels, as well as improved post-prandial breakfast glucose in the supplement vs. control product [63].

The NIGOHealth study is the only one examining the effect of an LGI/SD product during pregnancy on maternal and infant outcomes in a European population of obese pregnant women. It is a moderately large study with 332 recruited women, 232 newborns, and a large set of demographical and clinical characteristics that permit adjusting for multiple confounding factors. Moreover, intervention took place earlier during the pregnancy, compared to other LGI diet studies [24], and for a longer period of time [42]. However, some limitations should be addressed. Unexpected baseline differences in glucose biomarkers, despite the randomization scheme undertaken in the NIGOHealth RCT, could have masked a potential effect of the SP. In fact, an important finding is that the frequency of GDM cases was higher in the IG than in the SOC group, a fact that might be explained by the differences in glucose metabolism parameters found at baseline. Nevertheless, a sub-analysis including only those participants who may not have a certain predisposition to eGDM, according to IADPSG recommendations (FBG < 92 mg/dL) [38], was performed. Results from this secondary analysis once again showed that AUC at V2 was statistically the same in both groups in either the Evaluable or ITT cohort. Similarly, for MFBG and neonatal body composition, results showed no differences between the groups. Nevertheless, maternal HbA1c at V2 and V3 were significantly lower for the IG compared with the SOC group in the Evaluable cohort. However, results should be taken with caution as the number of subjects involved in that analysis is limited. In addition, the analysis comparing trajectories of MFBG, HOMA-IR, insulin, and HbA1c showed similar patterns. This within-group analysis would not prove an added benefit of the SP in the IG, as the significance between-group differences for primary outcomes has been established, but a potential effect of the SP cannot be ruled out.

As is typical in studies where one of the arms provides oral supplementation and one does not, the main difference in reasons for non-evaluability between groups revolves around non-compliance to the feeding regimen, acceptability, and tolerability. As nausea and emesis are a common pregnancy effect, it is probable that the pregnancy itself may have caused more tolerance issues than usual. Study dropouts have the potential to disrupt the balance of risk factors at randomization and diminish the sample size resulting in bias or loss of power. The uneven rates of non-evaluability between groups are limitation of the study and warrant further investigation.

Moreover, in the ITT cohort, not all women enrolled in the IG consumed the same product amount, nor was the duration of treatment similar in all participants; nonetheless, data from the evaluable cohort guaranteed a minimum intake of 75% of the SP, enough to detect differences in the treatment. Another limitation is the lack of dietary intake collection; hence, the exclusion of these data in the analysis of the participants involved. However, as all the participants were obese, close control of GWG by physicians was achieved following international recommendations [64]. Nevertheless, since high GI diets, rather than normal diets, are the dominant ones in Western industrialized societies [43], it would be interesting to analyze the effects of total dietary intake in a well-powered designed study. Finally, as this cohort is mainly from the Spanish population, data derived from it would not be generalizable to other populations.

## 5. Conclusions

In summary, no significant differences between groups for maternal blood glucose AUC at 28 weeks of gestation in the NIGOHealth cohort of obese pregnant women were observed. However, in additional analysis considering the differences in glucose metabolism markers at baseline, a significant difference was observed in HbA1c at V3, being lower in the IG vs. SOC group. The evolutionary analysis of Evaluable participants showed a significant increase in HbA1c from V2 to V3 in the SOC group, but not in the IG, which might suggest a potential effect of the intervention on Evaluable participants. More studies should be carried out to explore the influence of LGI/LGL products in obese and at-risk pregnant women to prevent hyperglycemia and its consequences.

## Figures and Tables

**Table 1:** Schedule of the NIGOHealth RCT assessments.

Assessment	Screening ≤ 14^+6^ Weeks	Visit 1/Baseline 15^+0^–16^+6^ Weeks	Visit 2 27^+0^–28^+6^ Weeks	Visit 3 34^+0^–36^+0^ Weeks	Visit 4 Parturition
Verify Eligibility	√	√			
Informed consent(s)	√				
Pre-pregnancy BMI	√				
Subject medical and family history, demographic data	√				
SP taste test	√				
Socioeconomic information	√				
Current medication(s)/supplement(s)	√	√	√	√	
Randomization		√			
Anthropometry		√	√	√	
Pregnancy/maternal health		√	√	√	
Fetal ultrasounds		√	√	√	
QoL questionnaires (EQ-5D, IPAQ, EPDS, FFQ)		√	√	√	
Fasting blood samples ^1^		√	√	√	
OGTT (75 g, 2 h)		√	√		
GDM diagnosis			√		
Distribution of SP, nutritional information, and Product Intake Records (IG only)		√	√	√	
Palatability questionnaire (IG only)			√	√	
Delivery and newborn data					√
Infant PEA POD^®^ and anthropometry					√
Umbilical cord blood ^2^					√
Umbilical cord tissue					√
Placental tissue ^2^					√
Adverse Events		√	√	√	√
Maternal blood at delivery ^1,2^					√
Colostrum sample					√
Infant meconium and buccal cheek swab					√

BMI: Body Mass Index; QoL: Quality of Life; EQ-5D: EuroQol 5-Dimension; IPAQ: International Physical Activity Questionnaire; EPDS: Edinburgh Postnatal Depression Scale; FFQ: Food Frequency Questionnaire; OGTT: Oral Glucose Tolerance Test; GDM; Gestational Diabetes Mellitus; IG: Intervention Group; SP: Study Product. ^1^ Metabolomic analysis; ^2^ Epigenetic analysis.

**Table 2:** Demographic and baseline characteristics of the NIGOHealth obese pregnant women.

Demographic and Lifestyle Parameters		IG (*n* = 230)	SOC (*n* = 102)	*p*
Maternal Age		31.43 ± 0.34	30.39 ± 0.56	0.10
Parity	0	45.2% (104)	45.1% (46)	0.12
	1	43.9% (101)	36.3% (37)	
	>1	10.9% (25)	18.6% (19)	
Main Ethnicity	German	0.9% (2)	1% (1)	0.82
	Spanish	91.7% (211)	90.2% (92)	
	South American	2.6% (6)	4% (4)	
	Arabic	2.2% (5)	3.9% (4)	
	African	0.4% (1)	0% (0)	
	Other	2.2% (5)	1% (1)	
Current Job Situation	Going to school or college full-time	3% (7)	2% (2)	**<0.001**
	In paid employment or self-employed	55% (127)	40% (41)	
	On a government employment	2% (4)	8% (8)	
	Looking after home or family	40% (92)	46% (47)	
	Doing something else	0% (0)	4% (4)	
Highest Educational Level	<High school/secondary school	13% (29)	20% (20)	0.071
	High school equivalent	12% (27)	7% (7)	
	High school	20% (47)	15% (15)	
	Some college/Professional training	28% (64)	36% (37)	
	University: Diploma course/Degree	22% (51)	22% (22)	
	Postgraduate (Master, PhD.)	5% (12)	1% (1)	
Total Years of Full-Time Education		14.00 (12.00, 17.00)	13.00 (10.00, 15.00)	0.057
Smoking	Never	55% (127)	50% (51)	0.63
	Ex-gave up before pregnancy	20% (46)	25% (26)	
	Ex gave up during pregnancy	9% (20)	7% (7)	
	Currently	16% (37)	18% (18)	
Alcohol (per week) consumed pre-pregnancy		0.00 (0.00, 1.00)	0.00 (0.00, 1.00)	0.74
Maternal parameters at Baseline/Visit 1				
Weight (kg)		91.99 ± 0.88	92.47 ± 1.16	0.75
Height (cm)		1.63 ± 0.004	1.63 ± 0.006	0.73
BMI (kg/m^2^)		34.47 ± 0.26	34.84 ± 0.39	0.47
	BMI 30–34.99 (kg/m^2^)	65.7% (151)	61.8% (63)	0.64
	BMI 35–39.99 (kg/m^2^)	25.7% (59)	29.4% (30)	
	BMI ≥ 40 (kg/m^2^)	8.7% (20)	8.8% (9)	
Triceps SF (mm)		34.26 ± 0.47	33.19 ± 0.68	0.21
Biceps SF (mm)		25.72 ± 0.46	25.25 ± 0.65	0.56
Subscapular SF (mm)		35.80 ± 0.55	35.17 ± 0.99	0.55
Suprailiac SF (mm)		41.90 ± 0.66	40.52 ± 1.14	0.27
Arm Diastolic Blood Pressure (mm Hg)		62.20 ± 0.53	64.59 ± 0.76	**0.012**
Pulse (beats/min)		85.85 ± 0.66	84.43 ± 1.01	0.24
Glucose (mg/dL)		84.71 ± 0.60	80.45 ± 0.98	**<0.001**
Cholesterol (mg/dL)		203.68 ± 2.14	205.86 ± 3.52	0.58
Triglycerides (mg/dL)		135.93 ± 2.84	139.47 ± 4.99	0.51
Hemoglobin A1c (%)		5.140 ± 0.017	5.089 ± 0.025	0.097
Insulin (µU/mL)		11.52 ± 0.36	10.29 ± 0.42	**0.044**
HOMA-IR		2.446 ± 0.087	2.071 ± 0.094	**0.010**
AUC		13,516.54 ± 210.53	13,073.08 ± 291.99	0.22
AAUC		3415.00 ± 183.47	3453.08 ± 271.48	0.91

Data are presented as mean ± Standard Error of the Mean (SEM) for parametrically analyzed data, % (*n*) for categorical data, and median (interquartile ranges) for non-parametrically analyzed data. ANOVA for normally distributed variables, Wilcoxon rank sum test for non-normal continuous variables, and Chi-square or Fisher test for categorical variables. Statistically significant p values (*p* < 0.05) are shown in **bold**. AUC Area Under Curve for maternal blood glucose; AAUC: Adjusted AUC; BMI: Body Mass Index; IG: Intervention Group; HOMA-IR: Homeostatic Model Assessment for Insulin Resistance; *p*: *p*-value; SF: Skinfold; SOC: Standard of Care.

**Table 3:** Comparison of glucose markers between the study groups at Visit 2 and 3 for the Evaluable cohort.

Visit	Variable	Evaluable			
		IG (*n* = 119)	SOC (*n* = 76)	*p*	*p* *
2	MFBG (mg/dL)	85.82 ± 0.80	82.20 ± 0.96	0.05	0.48
	HOMA-IR	2.96 ± 0.17	2.53 ± 0.12	0.072	0.91
	Insulin (µU/mL)	13.62 ± 0.65	12.46 ± 0.59	0.22	0.79
	HbA1c (%)	5.084 ± 0.026	5.100 ± 0.036	0.72	0.064
3	MFBG (mg/dL)	83.39 ± 0.83	80.21 ± 0.95	**0.015**	0.13
	HOMA-IR	2.94 ± 0.13	2.76 ± 0.16	0.39	0.49
	Insulin (µU/mL)	14.12 ± 0.56	13.73 ± 0.75	0.70	0.26
	HbA1c (%)	5.26 ± 0.03	5.31 ± 0.04	0.31	**0.007**

Values are expressed as Mean ± SEM. ANOVA for normally distributed variables. * ANCOVA with covariate maternal HbA1c and outcome itself at baseline, factor: Study Group, and covariates: maternal ethnicity, age, BMI, parity. Statistically significant *p* values (*p* < 0.05) are shown in **bold**. HOMA-IR: Homeostatic Model Assessment for Insulin Resistance; HbA1c: glycosylated hemoglobin IG: Intervention Group; MFBG: Maternal Fasting Blood Glucose; *p*: *p*-value; SOC: Standard of Care.

**Table 4:** Variation in curve slopes * for the Evaluable cohort.

Variable *	IG	SOC	*p*_adj_
MFBG (mg/dL)	−1.3845 ± 0.5057	−0.1521 ± 0.6067	0.12
Insulin (µU/mL)	0.9927 ± 0.2662	1.7406 ± 0.3265	0.077
HOMA-IR	0.1609 ± 0.0640	0.3383 ± 0.0784	0.081
HbA1c (%)	0.0500 ± 0.0121	0.1093 ± 0.0154	**0.0028**

* Model adjusted for age, BMI, ethnicity, and parity. Statistically significant *p* values (*p* < 0.05) are shown in **bold**. MFBG: Maternal Fasting Blood Glucose, HbA1c: glycosylated hemoglobin; IG: Intervention group; HOMA-IR: Homeostatic Model Assessment for Insulin Resistance; *p*_adj_: adjusted *p*-value; SOC: Standard of Care.

**Table 5:** Neonatal body composition (PEA POD^®^) and anthropometry ≤ 48 h after birth in newborns of the NIGOHealth study.

Cohort	ITT				Evaluable			
PEA POD^®^ Parameters	IG (*n* = 77)	SOC (*n* = 49)	*p*	*p*_adj_	IG (*n* = 35)	SOC (*n* = 47)	*p*	*p*_adj_
Fat (%)	9.70 ± 0.54	8.33 ± 0.57	0.098	0.14	9.71 ± 0.76	8.17 ± 0.58	0.11	0.22
FM (kg)	0.33 ± 0.02	0.27 ± 0.02	**0.047**	0.065	0.34 ± 0.03	0.26 ± 0.02	**0.034**	0.095
FFM (%)	90.31 ± 0.54	91.73 ± 0.57	0.085	0.12	90.29 ± 0.76	91.89 ± 0.59	0.094	0.20
FFM (kg)	2.876 ± 0.054	2.881 ± 0.051	0.96	0.98	2.95 ± 0.09	2.89 ± 0.05	0.53	0.82
Body mass (kg)	3.25 ± 0.05	3.15 ± 0.07	0.17	0.17	3.35 ± 0.08	3.15 ± 0.06	**0.039**	0.10
Body Volume (L)	3.11 ± 0.05	2.96 ± 0.07	0.056	0.068	3.20 ± 0.08	2.96 ± 0.07	**0.023**	0.078
Body density (kg/L)	1.047 ± 0.001	1.050 ± 0.001	0.056	0.087	1.047 ± 0.001	1.049 ± 0.001	0.12	0.26
FM density (kg/L)	0.8964 ± 0.0043	0.9007 ± 0.00	1	-	0.9007 ± 0.00	0.9007 ± 0.00	1	-
FFM density (kg/L)	1.090 ± 0.0003	1.065 ± 0.0001	0.57	0.63	1.065 ± 0.0005	1.065 ± 0.0001	0.41	0.50
Body surface area (cm^2^)	2223.88 ± 19.64	2182.80 ± 25.45	0.20	0.22	2262.58 ± 30.55	2186.35 ± 26.03	0.061	0.16
Thoracic gas volume (L)	0.144 ± 0.038	0.122 ± 0.019	0.67	0.55	0.191 ± 0.083	0.123 ± 0.020	0.37	0.27
**Anthropometry**	**IG (*n* = 144)**	**SOC (*n* = 71)**	***p***	***p*** **_adj_**	**IG (*n* = 72)**	**SOC (*n* = 65)**	***p***	***p*** **_adj_**
Weight (g)	3368.88 ± 36.78	3239.19 ± 58.35	0.053	0.10	3417.27 ± 50.54	3268.81 ± 56.48	0.051	0.18
Length (cm)	49.83 ± 0.20	49.33 ± 0.31	0.17	0.27	50.06 ± 0.25	49.50 ±0.29	0.15	0.60
HC (cm)	32.54 ± 0.16	31.86 ± 0.26	0.15	0.21	34.73 ± 0.23	31.88 ± 0.27	**0.045**	0.22
AC (cm)	32.54 ± 0.16	31.86 ± 0.26	**0.021**	**0.021**	32.73 ± 0.23	31.88 ± 0.27	**0.017**	0.074
SF Biceps (mm)	4.64 ± 0.12	4.90 ± 0.24	0.27	0.10	4.67 ± 0.18	4.88 ± 0.26	0.51	0.20
SF Triceps (mm)	5.41 ± 0.12	5.44 ± 0.21	0.91	0.59	5.34 ± 0.17	5.31 ± 0.20	0.91	0.61
SF Subscapular (mm)	5.03 ± 0.10	5.01 ± 0.17	0.94	0.69	4.95 ± 0.12	4.93 ± 0.17	0.91	0.56
SF Suprailiac (mm)	4.08 ± 0.09	3.97 ± 0.12	0.47	0.94	4.13 ± 0.12	3.94 ± 0.12	0.27	0.67

Data are expressed as Mean ± SME (Standard Error of the Mean). ANOVA for normally distributed variables. ANCOVA with covariates maternal HbA1c at baseline, maternal ethnicity, age, BMI, and parity. Statistically significant *p* values (*p* < 0.05) are shown in **bold**. AC: Abdominal Circumference FM: Fat Mass; FFM: Fat-Free Mass; HC: Head Circumference; IG: Intervention Group; ITT: Intention-to-Treat; IG: Intervention Group; *p*_adj_: adjusted *p*-value; *p*: *p*-value; SF: Skinfold; SOC: Standard of Care.

**Table 6:** Variables analyzed for participants with MFBG < 92 mg/dL at V1. Results showed for Evaluable cohort.

	Time Point	Variable	*n*	IG	*n*	SOC	*p*
Mother	V2	Total AUC	49	14,339.39 ± 365.06	37	14,211.89 ± 399.09	0.17
		AAUC	49	4484.69 ± 326.99	37	4410.81 ± 385.67	0.35
		MFBG (mg/dL)	90	83.46 ± 0.82	72	81.58 ± 0.93	0.72
		Insulin (µU/mL)	90	12.67 ± 0.56	72	12.37 ± 0.61	0.73
		HOMA-IR	90	2.641 ± 0.135	72	2.490 ± 0.125	0.95
		HbA1c (%)	84	5.06 ± 0.03	55	5.10 ± 0.04	**0.022**
	V3	MFBG (mg/dL)	86	81.74 ± 0.92	68	80.37 ± 0.98	0.49
		Insulin (µU/mL)	85	13.51 ± 0.60	68	13.88 ± 0.78	0.20
		HOMA-IR	85	2.73 ± 0.13	67	2.79 ± 0.16	0.28
		HbA1c (%)	78	5.24 ± 0.04	60	5.31 ± 0.04	**0.003**
Neonate	PEA POD^®^	Fat (%) *	26	9.54 ± 0.96	45	8.11 ± 0.61	0.27
		FM (kg) *	26	0.33 ± 0.04	45	0.26 ± 0.02	0.11
		FFM (%) *	26	90.46 ± 0.96	45	91.96 ± 0.61	0.24
		FFM (kg) *	26	2.99 ± 0.055	45	2.89 ± 0.053	0.23
		Body mass (kg) *	26	3.32 ± 0.09	45	3.15 ± 0.06	0.11
		Body Volume (L) *	26	3.17 ± 0.09	45	2.96 ± 0.07	0.10
		Body density (kg/L) *	26	1.047 ± 0.002	45	1.049 ± 0.001	0.29
		FM density (kg/L) *	26	0.9007 ± 0.0000	45	0.9007 ± 0.0000	-
		FFM density (kg/L) *	26	1.064 ± 0.001	45	1.065 ± 0.000	0.31
		Body surface area (cm^2^) *	26	2253.75 ± 35.21	45	2187.16 ± 26.75	0.15
		Thoracic gas volume (L) *	26	0.22 ± 0.11	45	0.12 ± 0.02	0.21
	Anthropometry	Weight (g) *	52	3371.06 ± 62.61	64	3253.13 ± 56.32	0.17
		Length (cm) *	50	50.01 ± 0.29	63	49.51 ± 0.30	0.42
		HC (cm) *	50	34.77 ± 0.40	63	34.01 ± 0.20	0.15
		AC (cm) *	50	32.70 ± 0.26	60	31.84 ± 0.28	0.08
		SF Biceps (mm) *	50	4.83 ± 0.22	61	4.93 ± 0.26	0.47
		SF Triceps (mm) *	50	5.44 ± 0.20	61	5.32 ± 0.21	0.91
		SF Subscapular (mm) *	50	5.01 ± 0.14	61	4.88 ± 0.17	0.87
		SF Suprailiac (mm) *	49	4.28 ± 0.15	59	3.93 ± 0.13	0.16

Values are expressed as Mean ± SME. ANOVA (Study Group, HbA1c, X variable at V1, Age, BMI, Ethnicity, Parity); * ANCOVA (Study Group, HbA1c, Age, BMI, Ethnicity, Parity). Statistically significant *p* values (*p* < 0.05) are shown in **bold**. AC: Abdominal Circumference; AUC: Area Under the Curve, AAUC: Adjusted AUC; FM: Fat Mass; FFM: Fat-Free Mass; HC: Head Circumference; HOMA- IR: Homeostatic Model Assessment for Insulin Resistance, HbA1c: glycosylated hemoglobin; IG: Intervention group; MFBG: Maternal Fasting Blood Glucose, *p*: *p*-value; SF: Skinfold; SOC Standard of Care.

## Data Availability

The data presented in this study are available on request from the corresponding author due to privacy and ethical restrictions.

## Supplementary Materials

The following supporting information can be downloaded at: https://www.mdpi.com/article/10.3390/nu17111942/s1, Supplementary Tables S1–S5: Table S1: Approximate study product composition; Table S2: Maternal and neonatal characteristics of the NIGOHealth study; Table S3: Baseline maternal glucose parameters (glucose, insulin, and HOMA) in subjects with GDM and Non-GDM diagnosed; Table S4: Effects of nutritional intervention with the LGI/SD product on maternal (A), and offspring anthropometry (B) and PEA POD^®^ ≤ 48 h body composition (C) outcomes depending on GDM condition; Table S5: AEs and SAEs in NIGOHealth RCT.
