# Supplementary material for: Effect of a Low Glycemic Index/Slow Digesting (LGI/SD) Carbohydrate Product on Maternal Glycemia and Neonatal Body Composition in Obese Pregnant Women: The NIGOHealth Randomized Clinical Trial
Source: Nutrients. 2025 Jun 5;17(11):1942. doi: 10.3390/nu17111942 (PMC12156999; doi:10.3390/nu17111942)
Supplement: Supplementary file 1 [file nutrients-17-01942-s001.zip › nutrients-3602910-supplementary.pdf]

Effect of a low glycemic index/slow digesting (LGI/SD) carbohydrate product on maternal glycemia and neonatal body composition in obese pregnant women: The NIGOHealth Randomized Clinical Trial.

Supplementary Tables

Supplementary Table S1. Approximate study product composition.

	Units	Per Serving (237 ml)	% of Total Energy
Energy	kcal	152	
Protein	g	7	18
Fat	g	3.5	21
Carbohydrate blend	g	18.4	48
Rapid digesting carbohydrate	g	1.94	
Slow digesting carbohydrate	g	16.46	
Fiber	g	6.2	13
Indigestible fiber	g	0.85	
Digestible fiber	g	5.35	
Vitamin A	IU	405	
Vitamin C	mg	70	
Calcium	mg	250	
Iron	mg	6.1	
Vitamin D	IU	200	
Vitamin E	IU	14.2	
Vitamin K	µg	16.2	
Thiamin (B1)	mg	0.77	
Riboflavin (B2)	mg	0.84	
Niacin	mg	5.2	
Vitamin B6	mg	1.0	
Folic acid	µg	300	
Vitamin B12	µg	2.2	
Biotin	µg	4.4	
Pantothenic Acid	mg	3.0	
Sodium	mg	102	
Potassium	mg	515	
Phosphorus	mg	476	
Iodine	µg	22	
Magnesium	mg	58	
Zinc	mg	10	
Selenium	µg	32	
Copper	mg	0.5	
Manganese	mg	0.6	

Chromium	µg	30	
Chloride	mg	200	
Choline	mg	120	

Supplementary Table S2. Maternal and neonatal characteristics of the NIGOHealth study.

Variables Analyzed		ITT (n=232)			Evaluable (n=145)		
		IG (n=155)	SOC (n=77)	*p*	IG (n=76)	SOC (n=69)	*p*
**Mother**							
Weight (Kg) at V3		98.06±0.97	100.14±1.49	0.23	97.12±1.36	100.73±1.60	0.086
GWG (Kg) at V3		7.22±0.42	7.48±0.59	0.72	6.14±0.59	7.57±0.62	0.10
GA at delivery (weeks)		39.17±0.11	39.18±0.17	0.97	39.24±0.15	39.38±0.15	0.50
Mode of delivery	Vaginal	62.6% (97)	70.1% (54)	0.44	61.8% (47)	71.0% (49)	0.36
	C-section	36.8% (57)	29.9% (23)		36.8% (28)	29.0% (20)	
	Other	0.6% (1)	0% (0)		1.3% (1)	0% (0)	
**Neonate**							
Prematurity		5.2% (8)	3.9% (3)	0.67	NA	NA	-
SGA		2.6% (4)	3.9% (3)	0.57	0.0% (0)	2.9% (2)	0.13
LGA		9.0% (14)	5.3% (4)	0.32	7.9% (6)	5.9% (4)	0.64
Sex	Boy	52.9% (82)	63.6% (49)	0.12	48.7% (37)	62.3% (43)	0.10
	Girl	47.1% (73)	36.4% (28)		51.3% (39)	37.7% (26)	
APGAR	1 min	9 (7)	9 (3)	0.52	9 (0)	9 (0)	0.33
	5 min	9 (5)	9 (2)	0.79	10 (1)	9 (1)	0.35

Data are expressed as Mean ± SME for parametrically distributed data, n (%) for categorical data, and median (IQR, interquartile ranges) for non-parametrically distributed data. ANOVA for normally distributed variables, Kruskal Wallis test and Wilcoxon rank sum test for non-normal continuous variables, and Chi-square or Fisher test for categorical variables. AC: Abdominal Circumference; GA: gestational age; GWG: Gestational Weight Gain; HC: Head Circumference; LGA: Large for gestational age; NA: Not Applicable; SGA: Small for gestational age; *p*: p-value.

Supplementary Table S3. Baseline maternal glucose parameters (glucose, insulin, and HOMA) in subjects with GDM and Non-GDM diagnosed.

Parameter	GDM (n=53)	Non GDM (n=186)	*p*
Glucose (mg/dL)	89.64±1.08	81.63±0.66	**<0.001**
Insulin (μU/mL)	12.74±0.77	10.58±0.36	**0.007**
HOMA-IR	2.85±0.19	2.15±0.08	**<0.001**
HbA1c (%)	5.217±0.033	5.108±0.018	**0.004**

Values are expressed as Mean±SEM. ANOVA for normally distributed variables, GDM: Gestational Diabetes Mellitus; HbA1c: glycosylated hemoglobin; HOMA- IR: Homeostatic Model Assessment for Insulin Resistance.

Supplementary Table S4. Effects of nutritional intervention with the LGI/SD product on maternal (A), and offspring anthropometry (B) and PEA POD®≤48 h body composition (C) outcomes depending on GDM condition.

A.

	ITT											
	**GDM**						**Non- GDM**					
**Outcome**	**n**	**IG**	**n**	**SOC**	***p***	***p*_adj**	**n**	**IG**	**n**	**SOC**	***p***	***p*_adj**
Total AUC V2	25	17452.80±394.05	7	18235.71±730.06	0.36	0.19	64	13520.16±214.23	34	13616.47±299.97	0.79	0.52
AAUC V2	25	6484.80±555.98	7	6955.71±829.33	0.63	0.62	64	3824.53±199.84	34	4012.94±338.34	0.61	0.37
MFBG V2	48	93.60±1.27	12	90.92±3.22	0.37	0.66	111	81.62±0.61	63	80.35±0.82	0.21	0.42
Insulin (μU/mL) V2	48	16.46±1.38	12	11.21±.0.95	0.068	0.50	111	11.85±0.41	63	12.66±0.68	0.29	0.11
HOMA-IR V2	48	3.89±.0.37	12	2.53±.0.24	0.077	0.91	111	2.41±0.09	63	2.51±0.14	0.49	0.24
HbA1c (%) V2	45	5.17±0.05	8	5.13±0.07	0.72	0.82	98	5.04±0.03	50	5.10±0.04	0.19	**0.046**
MFBG V3	48	87.19±1.39	12	83.42±3.15	0.24	0.42	105	81.22±0.78	59	79.56±0.94	0.19	0.23
Insulin (μU/mL) V3	47	14.48±0.98	11	11.65±0.65	0.18	0.95	103	14.03±0.57	60	14.15±0.87	0.90	0.52
HOMA-IR V3	47	3.17±0.24	11	2.39±0.16	0.13	0.85	102	2.85±0.12	59	2.83±0.18	0.95	0.74
HbA1c (%) V3	45	5.36±0.06	9	5.36±0.09	0.97	0.79	93	5.22±0.03	54	5.30±0.04	0.092	**0.018**
	**Evaluable**											
	**n**	**IG**	**n**	**SOC**	***p***	***p*_adj**	**n**	**IG**	**n**	**SOC**	***p***	***p*_adj**
Total AUC V2	17	17961.18±481.48	7	18235.71±730.06	0.76	0.17	41	13494.88±280.65	34	13616.47±299.97	0.77	0.081
AAUC V2	17	6949.41±555.98	7	6955.71±829.33	0.99	0.78	41	3833.41±265.91	34	4012.94±338.34	0.67	0.15
MFBG V2	37	94.22±1.24	12	90.92±3.22	0.25	0.95	80	81.84±0.67	63	80.35±0.82	0.16	0.99
Insulin (μU/mL) V2	37	17.35±1.65	12	11.21±0.95	**0.044**	0.29	80	11.77±0.47	63	12.66±0.68	0.27	**0.038**
HOMA-IR V2	37	4.12±0.45	12	2.53±0.24	0.055	0.57	80	2.39±0.10	63	2.52±0.14	0.46	0.092
HbA1c (%) V2	35	5.19±0.05	8	1.23±.0.07	0.60	0.93	75	5.04±0.03	50	5.10±0.04	0.18	**0.017**
MFBG V3	37	87.00±1.64	12	83.42±3.15	0.30	0.52	77	81.65±0.88	59	79.56±0.94	0.11	0.31
Insulin (μU/mL) V3	36	14.34±1.13	11	11.65±0.65	0.20	0.90	76	14.02±0.63	60	14.15±0.87	0.90	0.28
HOMA-IR V3	36	3.16±0.29	11	2.39±0.16	0.16	0.95	76	2.83±0.14	59	2.83±0.18	0.99	0.40
HbA1c (%) V3	38	5.35±0.06	9	5.36±0.09	0.95	0.68	69	5.22±0.03	54	5.30±0.03	0.10	**0.002**

B.

Anthropometry	ITT*											
	GDM						Non- GDM					
	**n**	**IG**	**n**	**SOC**	p	p_{adj}	**n**	**IG**	**n**	**SOC**	p	p_{adj}
Weight (g)	47	3450.32±62.55	12	3434.17±167.09	0.92	0.85	105	3335.84±45.34	59	3198.47±63.55	0.076	0.056
Length (cm)	43	49.75±0.45	10	49.74±0.71	0.99	0.88	100	49.87±0.22	58	49.30±0.34	0.15	0.17
HC (cm)	43	34.77±0.44	10	34.16±0.31	0.52	0.77	100	34.25±0.18	58	33.93±0.24	0.29	0.25
AC (cm)	40	32.78±0.84	10	32.07±0.83	0.38	0.31	89	32.42±0.18	53	31.83±0.28	0.067	0.065
Biceps SF (mm)	40	4.60±0.21	10	4.81±0.51	0.66	0.74	89	4.63±0.15	54	4.90±0.29	0.36	0.19
Triceps SF (mm)	40	5.68±0.24	10	6.10±0.63	0.47	0.53	89	5.24±0.14	54	5.20±0.21	0.90	0.97
Subscapular SF (mm)	40	5.09±0.17	10	5.91±0.47	**0.047**	0.058	89	5.00±0.12	54	4.76±0.17	0.23	0.26
Suprailiac SF (mm)	40	4.08±0.13	10	4.30±0.21	0.44	0.39	88	4.07±0.11	52	3.88±0.14	0.30	0.40
	Evaluable*											
	**n**	**IG**	**n**	**SOC**	p	p_{adj}	**n**	**IG**	**n**	**SOC**	p	p_{adj}
Weight (g)	28	3529.82±73.76	12	3434.17±169.09	0.55	0.89	47	3250.21±66.27	55	3232.73±57.83	0.18	0.22
Length (cm)	27	50.36±0.31	10	49.74±0.71	0.36	0.67	45	49.88±0.35	55	49.46±0.33	0.38	0.51
HC (cm)	27	35.26±0.65	10	34.16±0.31	0.32	0.58	45	34.42±0.25	55	33.99±0.23	0.20	0.25
AC (cm)	25	33.10±0.40	10	32.07±0.83	0.22	0.32	44	32.52±0.27	52	31.84±0.29	0.09	0.16
Biceps SF (mm)	25	4.74±0.31	10	4.81±0.51	0.91	0.89	44	4.63±0.22	53	4.89±0.29	0.50	0.21
Triceps SF (mm)	25	5.67±0.32	10	6.10±0.63	0.51	0.48	44	5.16±0.18	53	5.16±0.21	0.97	0.77
Subscapular SF (mm)	25	5.11±0.22	10	5.91±0.48	0.09	0.11	44	4.86±0.14	53	4.74±0.17	0.61	0.72
Suprailiac SF (mm)	25	4.14±0.16	10	4.30±0.21	0.57	0.50	43	4.13±0.17	51	3.87±0.14	0.24	0.34

C.

PeaPod ≤48 h	n	IG	n	SOC	*p*	p_{adj}	n	IG	n	SOC	*p*	p_{adj}
	**ITT***											
	**GDM**						**Non- GDM**					
Fat (%)	26	10.83±0.94	4	8.70±2.31	0.41	0.56	50	9.27±0.65	43	8.13±0.61	0.21	0.14
FM (kg)	26	0.37±0.04	4	0.29±0.08	0.45	0.58	50	0.31±0.03	43	0.26±0.02	0.12	0.068
FFM (%)	26	89.17±0.94	4	91.30±2.31	0.41	0.56	50	90.73±0.65	43	91.94±0.61	0.18	0.12
FFM (kg)	26	2.95±0.06	4	3.02±0.05	0.67	0.57	50	2.84±0.08	43	2.88±0.06	0.71	0.81
Body Mass (kg)	26	3.32±0.08	4	3.31±0.11	0.97	0.87	50	3.22±0.06	43	3.13±0.06	0.31	0.22
Body Volume (L)	26	3.20±0.08	4	3.16±0.11	0.86	0.99	50	3.08±0.06	43	2.94±0.08	0.14	0.10
Body Density (kg/L)	26	1.045±0.002	4	1.049±0.004	0.40	0.54	50	1.047±0.001	43	1.050±0.001	0.21	0.14
FM Density (kg/L)	26	0.901±0.000	4	0.901±0.000	1	-	50	0.90±0.00	43	0.90±0.00	1	-
FFM Density (kg/L)	26	1.065±0.001	4	1.065±0.001	0.95	0.85	50	1.065±0.003	43	1.065±0.001	0.38	0.40
Body surface area (cm^2	26	2254.18±32.40	4	2252.03±51.22	0.98	0.85	50	2210.70±24.96	43	2180.24±27.99	0.42	0.29
Thoracic Gas Volume (L)	26	0.108±0.002	4	0.108±0.003	0.97	0.87	50	0.163±0.058	43	0.125±0.022	0.56	0.49
	**Evaluable***											
	**n**	**IG**	**n**	**SOC**	***p***	p_{adj}	**n**	**IG**	**n**	**SOC**	***p***	p_{adj}
Fat (%)	15	10.87±0.87	4	8.70±2.31	0.30	0.28	20	8.84±1.13	43	8.13±0.61	0.55	0.41
FM (kg)	15	0.38±0.04	4	0.29±0.09	0.31	0.25	20	0.31±0.05	43	0.26±0.02	0.27	0.18
FFM (%)	15	89.13±0.87	4	91.30±2.31	0.30	0.28	20	91.17±1.13	43	91.94±0.61	0.97	0.38
FFM (kg)	15	3.06±0.08	4	3.02±0.05	0.79	0.73	20	2.87±0.15	43	2.88±0.06	0.51	0.99
Body Mass (kg)	15	3.44±0.10	4	3.31±0.11	0.53	0.44	20	3.28±0.11	43	3.13±0.06	0.23	0.15
Body Volume (L)	15	3.29±0.10	4	3.16±0.11	0.51	0.41	20	3.12±0.44	43	2.94±0.49	0.16	0.11
Body Density (kg/L)	15	1.065±0.000	4	1.065±0.000	0.29	0.26	20	1.048±0.002	43	1.050±0.001	0.59	0.44
FM Density (kg/L)	15	0.901±0.000	4	0.901±0.000	1	-	20	0.901±0.000	43	0.901±0.000	1	-
FFM Density (kg/L)	15	1.065±0.001	4	1.065±0.001	0.95	0.82	20	1.064±0.001	43	1.065±0.000	0.22	0.18
Body surface area (cm^2	15	2299.77±38.76	4	2252.03±51.22	0.56	0.46	20	2234.69±44.68	43	2180.24±27.99	0.29	0.20
Thoracic Gas Volume (L)	15	0.111±0.026	4	0.108±0.003	0.55	0.44	20	0.251±0.145	43	0.125±0.022	0.22	0.19

Values are expressed as mean ± SEM. ANCOVA (Study Group, HbA1c, X variable at V1, Age, BMI, Ethnicity, Parity). *ANCOVA (Study Group, HbA1c, Age, BMI, Ethnicity, Parity). AC: Abdominal Circumference; AUC: Area under Curve; FM: Fat Mass; FFM: Fat Free Mass; GDM: Gestational Diabetes Mellitus; HC: Head Circumference; IG: Intervention Group; ITT: Intention to Treat; MFBG: Maternal Fasting Blood Glucose, SF: Skinfold; *p*: p-value; SOC: Standard of Care.

Supplementary Table S5. AEs and SAEs in NIGOHealth RCT.

	IG (n= 230)	SOC (n= 102)	Total (N = 332)
Adverse Events (AEs)			
**Anemia**	11 (4.8)	16 (15.7)	27 (8.1)
**Gastrointestinal disorders**	51 (22.2)	8 (7.8)	59 (17.8)
Diarrhea	11 (4.8)	0 (0.0)	11 (3.3)
Dyspepsia	26 (11.3)	7 (6.9)	33 (9.9)
Others (abdominal pain or discomfort, constipation, flatulence, …)	15 (6.5)	2 (2.0)	17 (5.1)
**Hypothyroidism**	2 (0.9)	2 (2.0)	4 (1.2)
**Hyperlipidemia**	16 (7.0)	13 (12.7)	29 (8.7)
**GDM**	52 (22.6)	13 (12.7)	65 (19.6)
**Pregnancy, puerperium and perinatal conditions**	9 (3.9)	4 (3.9)	13 (3.9)
Placental disorder	1 (0.4)	0 (0.0)	1 (0.3)
Pre-eclampsia	1 (0.4)	0 (0.0)	1 (0.3)
Premature delivery	0 (0.0)	2 (2.0)	2 (0.6)
Fetal growth restriction	1 (0.4)	2 (2.0)	3 (0.9)
Neonatal renal and urinary disorders	2 (0.9)	0 (0.0)	2 (0.6)
Neonatal respiratory distress	0 (0.0)	1 (1.0)	1 (0.3)
Neonatal hypoglycemia	1 (0.4)	0 (0.0)	1 (0.3)
**Surgical and medical procedures**	2 (0.9)	0 (0.0)	2 (0.6)
Abortion induced	1 (0.4)	0 (0.0)	1 (0.3)
Hospitalization	1 (0.4)	0 (0.0)	1 (0.3)
NICU admission	12 (5.2)	3 (2.9)	15 (4.5)
**Other AEs**	54 (23.5)	16 (15.7)	70 (21.1)
Severe Adverse Events (SAEs)			
Termination of pregnancy (tetralogy of Fallot)	1 (0.4)	0 (0.0)	1 (0.3)
Abortion late	0 (0.0)	1 (1.0)	1 (0.3)
HELLP syndrome	0 (0.0)	1 (1.0)	1 (0.3)
Neonatal bradycardia	1 (0.4)	0 (0.0)	1 (0.3)
Neonatal septic shock	1 (0.4)	0 (0.0)	1 (0.3)
Meconium aspiration syndrome	1 (0.4)	0 (0.0)	1 (0.3)
Neonatal asphyxia	2 (0.9)	0 (0.0)	2 (0.6)
Congenital anomaly	3 (1.3)	0 (0.0)	3 (0.9)
Neonatal death	1 (0.4)	0 (0.0)	1 (0.3)
